# Optimization of Grayscale Lithography for the Fabrication of Flat Diffractive Infrared Lenses on Silicon Wafers

**DOI:** 10.3390/mi15070866

**Published:** 2024-06-30

**Authors:** Angelos Bouchouri, Muhammad Nadeem Akram, Per Alfred Øhlckers, Xuyuan Chen

**Affiliations:** Department of Microsystems, University of South-Eastern Norway, Raveien 205, 3184 Borre, Norway; per.ohlckers@usn.no (P.A.Ø.); xuyuan.chen@usn.no (X.C.)

**Keywords:** grayscale, Fresnel lens, MEMS, infrared vision, multilevel, grayscale etching

## Abstract

Grayscale lithography (GSL) is an alternative approach to the standard binary lithography in MEMS fabrication, enabling the fabrication of complicated, arbitrary 3D structures on a wafer without the need for multiple masks and exposure steps. Despite its advantages, GSL’s effectiveness is highly dependent on controlled lab conditions, equipment consistency, and finely tuned photoresist (PR) exposure and etching processes. This works presents a thorough investigation of the challenges of GSL for silicon (Si) wafers and presents a detailed approach on how to minimize fabrication inaccuracies, aiming to replicate the intended design as closely as possible. Utilizing a maskless laser writer, all aspects of the GSL are analyzed, from photoresist exposure parameters to Si etching conditions. A practical application of GSL is demonstrated in the fabrication of 4-μm-deep f#/1 Si Fresnel lenses for long-wave infrared (LWIR) imaging (8–12 μm). The surface topography of a Fresnel lens is a good case to apply GSL, as it has varying shapes and size features that need to be preserved. The final fabricated lens profiles show a good match with the initial design, and demonstrate successful etching of coarse and fine features, and demonstrative images taken with an LWIR camera.

## 1. Introduction

Grayscale lithography (GSL) offers the ability to fabricate arbitrary multilevel, three-dimensional (3D) structures in a single photolithographic step. The traditional default approach, binary lithography (BL), uses an on-off modulation in the exposing and etching steps. The PR is either fully exposed or not. During development, depending on whether the photoresist (PR) type is positive or negative, a part of it will be removed. During the etching step, the exposed area of the underlying wafer will be etched away, leaving behind an etched substrate. After removing the remaining PR, one will have a structure with two different depth levels, thus the “binary” in its name. To increase the number of height levels, the same process must be followed again with a new mask. This will increase the complexity of the fabrication process and the chances that accumulated errors will render the device unusable. 

During GSL, the PR is scanned pixel by pixel and exposed to a modulated dose of a laser beam, reaching varying depths in the PR. After development, a 3D-patterned PR is obtained. For the etching step, a fully anisotropic approach is required to preserve the relief’s geometry. The patterned PR must be etched into the substrate by etching the PR and the substrate simultaneously and with a controlled etch rate for both. The nature of GSL requires well-controlled steps, and any discrepancy from the desired conditions will result in fabrication errors that will be amplified by the subsequent steps, rendering the final device unfit for use. 

GSL is not a new concept. It has existed for over 30 years [1] and has attracted the interest of many research groups for the capabilities it offers. The ability of GSL to fabricate arbitrary 3D structures makes it a noteworthy technique, suitable for several applications. MEMS devices [2,3,4,5,6], microfluidic devices with variable dimension channels [7], mold creation for nanoimprint techniques [8], non-binary topography of many photonic devices, and more [9,10,11] make GSL a suitable fabrication technique. Microlenses [12,13,14], circular Dammann gratings [15], multispectral filters arrays [16], and spectrometer chips [17] have been fabricated using GSL. Hakkel et al. patterned flat polymeric layers to varying thickness on top of pixels to fine-tune their spectral responses individually [18]. Fresnel lenses for the LWIR and short-wave infrared have also been fabricated using GSL [19,20,21]. A thermal scanning probe has been used in grayscale lithography to achieve extremely high resolution with step heights of 6 nm and 32 nm step width [22]. 

One of the most important parameters to control GSL is the ratio of etch rates of Si over PR, named etch selectivity. Khazi et al. [23] explored different etching parameters for etch selectivity control and anisotropy. The selectivity intervals explored were relatively large, and the etching rate of the PR was low enough to extend the etching duration to more than 4 h for a 4-μm-deep Fresnel lens when using their proposed recipe for 1:1 etch selectivity. Nachmias et al. [24] used deep reactive ion etching (DRIE) and reactive ion etching (RIE) to fabricate silicon Fresnel lenses. In the RIE process, the ratio of SF_6_/O_2_ was not explored as it is an important aspect of GSL to control the selectivity. Additionally, they used a low selectivity of 0.55:1 to compress the Si etch depth to his desired height of 600 nm, which is much less than needed for infrared lenses in our case. Yi-Hsiang Huang et al. used a digital micromirror device to create a mask pattern. The design consisted of just three grayscale levels [3]. Loomis et al. [25] and LeCompte et al. [26] approached the linearization of the PR behavior against exposure dose but their approach was slightly more complicated. Kaste et al. analyzed the behavior of PR and its relation to developer concentration, soft baking and development duration, and energy dosage but the resulting patterned PR had a high surface roughness and it was a single continuous slope [27]. Dunkel et al. linearized the PR’s behavior and fabricated lenses and blazed grating but their features’ dimensions were far larger than the ones that exist in the LWIR Fresnel lens [28]. For example, the blazed grating that can be similar to the lens’ rings, has a width of 800 μm and the lenses were not of a Fresnel type. Similarly for [29], the lateral periodicity of the gratings was 1 mm. Overall, even though GSL has been the subject of research of various groups, it has always been treated in a more isolated approach, meaning that the various issues that are present in GSL are not treated collectively. 

For our case study, we examined various aspects of GSL to accurately fabricate monochromatic Si Fresnel lenses, designed for use in LWIR imaging optical systems. The surface topography of a Fresnel lens consists of concentric rings of varying widths, having the same maximum height and a cross-sectional profile that follows a specific phase profile equation (usually a parabolic or hyperbolic phase profile). In the lens, central rings are wider and “flatter,” and, as the ring’s distance from the center increases, they become narrower and steeper. The coexistence of these different features makes the Fresnel lens an appropriate and relatively complex structure to apply to GSL. This proved to be an issue for achieving uniform development but was eventually resolved. These different geometries can be interpreted as “standalone” structures that have different fabrication demands and must be taken into consideration for a successful device. 

The nature of the GSL imposes careful planning for each step. The steps that are common with BL can be divided into the following: design, exposing, and etching. In the design step, the photolithographic mask is created from a given phase profile. This can be achieved in many ways, depending on the software capabilities of the available equipment. It can be CAD models, GDS files, simple grayscale bitmaps, or tiff files. The photolithographic mask requires an adjustment step that will compensate for the non-linear response of the PR to the exposure dosage. Exposing and developing the PR also requires extensive study because this step will greatly impact the final device. Many parameters need to be tuned for their optimal values. All the parameters are cross-linked with each other, and the optimal combination must be determined. These parameters are laser writer exposure mode, soft baking conditions, exposure dosage range and other machine-related parameters, and finally, the PR development. In the etching step, one desires to transfer the patterned PR into the substrate as accurately as possible. Having a process that has controllable selectivity as well as full anisotropy is quite important. Finally, all features must survive the etching step, regardless of shape, size, or height. In this work, a detailed approach to how to treat each step is presented.

### Background of Grayscale Lithography

In GSL, a linear response of the exposure dosage and PR development depth is required to be able to accurately control the developed height of the PR. While BL makes use of high contrast RPs, GSL benefits from low contrast PRs that exhibit a more linear behavior. Although GSL-specific PR exists, its linearity still lacks perfection, and it will benefit from a calibration step during the grayscale mask design. There are many cases when GSL is used while the PR photochemical behavior is neglected, resulting in PR patterns that do not match the design file [4]. The thought process behind the linearization is simple. Since the exposure depth is continuous for a continuous exposure dosage, there will be a specific dosage required to achieve a specific exposure depth. Calculating and selecting a range of specific dosages can result in a custom exposure curve that can convert a nonlinear response to a linear one. 

**Figure 1:**
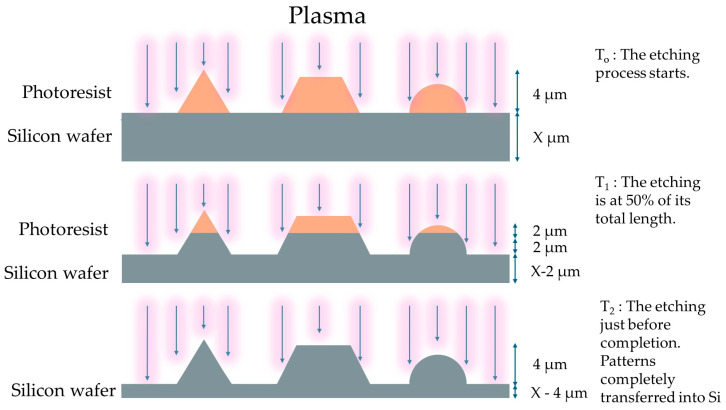
The evolution of the patterns during the etching step. The patterned PR and the exposed Si are etched simultaneously and anisotropically. The transfer of the patterns from the PR to the Si happens steadily throughout the etching cycle.

After exposure, consistent development of the device or wafer is required. All areas that share the same exposing dosage, according to the design, must have the same PR height, as it is crucial for the good imaging performance of the lens. However, it was observed that this was not the case. Additionally, any height differences will be amplified during the etching process when selectivity (Si:PR etching rates) is higher than 1:1, further degrading the quality of the lens. Etching is the last step of the GSL process and has its own requirements. The grayscale etching process must exhibit and maintain controlled behavior throughout its duration. Precise selectivity is needed to accurately convert the height of the patterned PR into patterned Si. Usually, a selectivity of 1:1 is sought after, as any etch scaling is generally not required, although there are cases where scaling is necessary, as presented later in this work. Anisotropy is equally important, as lateral etching will destroy or shrink features that are present on the wafer. For this purpose, a passivating gas is included in the etching gas chemistry. It will adhere to the wafer surface, but due to the vertical etching being more impactful due to ion bombardment, the passivation layer will be etched away from horizontal surfaces but will remain on the vertical sidewalls. Figure 1 shows how the patterned PR slowly transfers into the Si substrate. The etchant gases mix reacts equivalently with the PR and the Si substrate, slowly transferring the pattern into the wafer.

In our work, a Si Frensel lens was chosen as a case study because of its interesting surface topography, as previously explained. A monochromatic focusing lens’ phase is described by the following equation:

(1) $$\varphi \left(r\right)=\left(-\frac{2\pi }{\lambda }\right)\left(\sqrt{r^{2}+f^{2}}-f\right)$$

where *r* is the radial distance across the aperture, λ the operating wavelength, and *f* is the focal distance. The phase undergoes wrapping, keeping the values within the −π to π radian boundaries. The actual phase applied to radiation when traveling through a medium and surrounded by air is given by the following:

(2) $$\varphi \left(h\right)=h*\left(n_{s}-1\right)2\pi /\lambda$$

where *n_s_* is the refractive index of the substrate and *h* the etch depth of the medium. For Si with *n_s_* ~3.5 at 10 μm wavelength, an etch thickness of 4 μm is required to acquire a 2π phase.

## 2. Materials and Methods

### 2.1. Mask Design

The test structures design files were created using MATLAB (version: 9.14.0 (R2023a), The Mathworks Inc, Natick, MA, USA) and saved as 8-bit bitmap files. Each pixel value of the BMP files is represented by a value between 0 to 255, which will be referred to as “grayscale power levels”. For the lens design, a column text file starting from the center of the lens and ending at the edge of the lens was created in MATLAB. Then it was rotated around the center, creating a circularly symmetric lens. This step was performed using the software provided with the maskless writer, Picomaster 150i, with a 375 nm laser source (Raith Gmbh, Dortmund, Germany). Creating an entire 2D lens design on a bitmap file was not an option due to file size limitations that emerged. A pixel on the BMP file represents an actual area of the lens and a high-resolution mask was desired for an accurate profile design. Using the column approach, a densely pixelated lens was designed, with each “pixel” of the column matching the step size parameter of the maskless writer. Fresnel lenses of up to 2 cm in diameter with a pixel size of 225 nm were fabricated using this approach.

### 2.2. Linearization of the Photoresist

A GSL PR ma-P 1225G (Micro Resist Technology GmbH, Berlin, Germany) was used for the development of the linearization process for the PR and for obtaining the selectivity data. Subsequent experiments and final devices used a thicker variant, the 1275 G. The thinner PR (1225 G) was spin-coated (Spin Coater 150i from SPS-Europe B.V., Putten, The Netherlands) at 1000 rpm and soft-baked at 100 °C for 45 s. Then it was loaded onto the maskless writer. An initial step was to determine the appropriate dosage that would result in a 4 μm developed depth, measured with the profilometer Dektak 150 Stylus ProfilerScan (Veeco Instruments Inc, New York, NY, USA) and equipped with a 2.5 μm radius tip. An automated built-in feature was used for this purpose. The resolution mode of the maskless writer was set to a “medium resolution”, affecting the spot size, which is 550 nm in this case. The medium resolution mode has a better depth of focus, giving better exposure in the thick photoresist. The step size was set as half of the spot size, at 225 nm. The scanning speed was set at 300 mm/s, and the duty cycle was set to 6. After exposure, the PR was developed in mr-D 526/S developer (Micro Resist Technology GmbH, Berlin, Germany) for 45 s with gentle stirring. It was determined that a dose of around 225–235 mJ/cm^2^ is sufficient to reach the required depth in the PR. It must be noted that a new linearization is required whenever soft-baking conditions change, as the PR will behave and develop with a slightly different profile.

**Figure 2:**
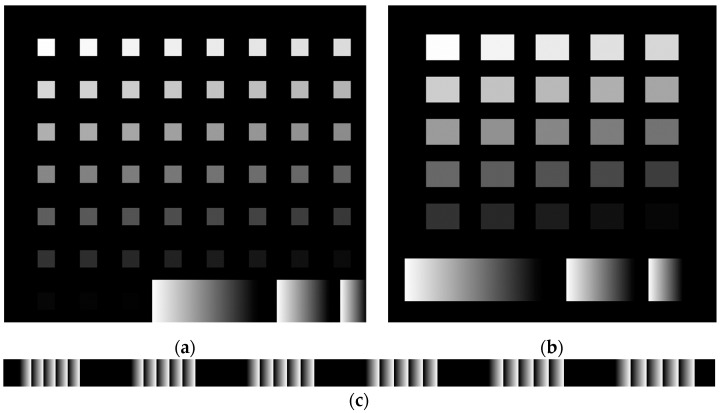
(**a**) The test structure for dose calibration and (**b**) the test structure for selectivity measurements are relatively identical structures. The calibration design has more squares, so the linearization is accurate, and the interpolation relies on a sufficient number of measurements. (**c**) Part of the test structure that is used to optimize the development process. The width of the ramps increases gradually. The structures will be referred to as test structures A, B, and C in the text.

For the linearization, test structure A, shown in Figure 2a, was used. It consists of 51 separated squares. The boxes’ grayscale power level decreases in value in steps of 5. The whitest areas are exposed to the maximum dosage, and the blackest to the minimum. The wafer was developed until the PR of the black area was fully removed from substrate, and the Si surface was exposed. Then the height of each individual box was measured and imported into MATLAB. The missing grayscale values between each square were interpolated, and then a polynomial fit was applied to minimize any big deviations. After obtaining the exposure curve, the actual calibration takes place.

The linearization algorithm retrieves the GS power values of the specific pixel “A” from the mask under optimization and the developed depth of the corresponding area. Also, by taking the minimum and maximum developed depths, a straight line connecting these two points is used as a reference for the ideal behavior. The algorithm continues and searches for a developed depth among the data that has the depth value that was expected from the specific pixel area. The closest match is selected, and the grayscale power value of that pixel “B” is retrieved and substituted for the value of the pixel “A”. This step is repeated for all the pixels of the mask.

### 2.3. Photolithography

#### 2.3.1. Exposing

Optimizing this step was found to be challenging. The initial parameters are mentioned in the previous section, but it was necessary to change them as it was observed that the development of the PR was not optimal, resulting in an etched structure that lacked accuracy. There was a height difference between the inner and outer rings of the Fresnel lens. A difference of up to 1.5 μm was observed, which is a 38% phase discrepancy between the designed and actual height. Such a height difference will result in wavefront aberrations and degrade the image quality of the fabricated lens. 

The initial photoresist was swapped for the thicker version, 1275 G. It was spin-coated at 6500 rpm and soft-baked for 3 min, resulting in a 5-μm-thick PR. A new test structure C was designed, shown in Figure 2c, consisting of sets of linear ramps with different widths, starting from 14 μm up to 140 μm. More specifically, 8 ramps from 14 to 21 μm widths, with increments of 1 μm; 23 to 37 μm widths, with increments of 2 μm; and 50 to 140 μm widths, with increments of 10 μm. It was also calibrated for the new PR. Two versions of it were created. One had gaps between each ramp, while the other had no gaps, simulating more closely the rings of a lens. For height measurements, the spaced version was used so that the profilometer could give accurate results. Both had several repetitions of each ramp in order to get a better overview of how ramps behave and to not rely on a single ramp measurement. Initial measurements showed that intermediary ramps, between the narrowest and widest ramps, behaved in an almost linear way. The narrower the ramp, the higher the height difference from the widest ramp. For that reason, we examined only the extremes. If that difference was reduced, so would the difference for the intermediary rings. This was confirmed by results presented later in this work. 

It was determined that, to have the least possible height difference between rings, exposure dosage and development time must change. A dosage of 155–165 mJ/cm^2^ was found to be the suitable range, with the development time being around 80 s. After exposure, the height difference between rings was measured to be around 0.2 μm, which is a substantial reduction from the initial 1.5 μm. The developed depth varied, ranging from 3.5 to 3.7 μm, which requires etching with a selectivity slightly higher than 1:1, so that the target 4 μm height in Si is reached. Furthermore, since the PR has a thickness of 5 μm, this leaves a layer of ~1.5 μm beneath the patterned PR, which will be required to etch away as well.

In Table 1, it can be seen that the height of narrow and wider ramps differed and was not consistent with development time. The height difference was best when the duration was 75 s but even then, the obtained height was not repeatable. Additionally, a relatively higher selectivity of 1.37:1 would be required to scale the structures to 4 μm. The preferred height range that was selected was 3.3 to 3.4 μm, requiring a selectivity of 1.2:1.

#### 2.3.2. Reactive Ion Etching—Parameter Optimization

A RIE process was chosen to transfer our patterns from PR into the Si. A constant flow of SF_6_, O_2_, and CHF_3_ was supplied in the etching chamber (100 Estrelas from Oxford Instruments, Bristol, United Kingdom). SF_6_ is mainly used to etch the Si, CHF_3_ for sidewall passivation, and O_2_ for the predominant etching of the PR and removal of the passivation from “flat” surfaces. CHF_3_ flow rate was set to 15 standard cubic centimeters per minute (sccm) and was kept constant throughout this study. The temperature was set to 5 °C, the pressure was 10 mTorr, and the radio frequency power was set to 150 watts. The solution for the non-uniform PR development involves higher selectivity and longer etching duration, as height scaling and additional PR thickness etching need to be addressed. 

Depending on the available materials, designs, and desired structure height, one may have to scale the PR pattern to the desired height by a different amount. For accurate scaling calibration, two sets of etching experiments were conducted. Initially, the parameters for selectivity 1:1 were determined. Then, one set kept the SF_6_ flow rate constant, while varying the O_2_ flow rate, and the opposite for the other set of experiments, examining the effect of the flow rates on selectivity. Flow rates with a selectivity of less than 1:1 were also examined.

**Figure 3:**
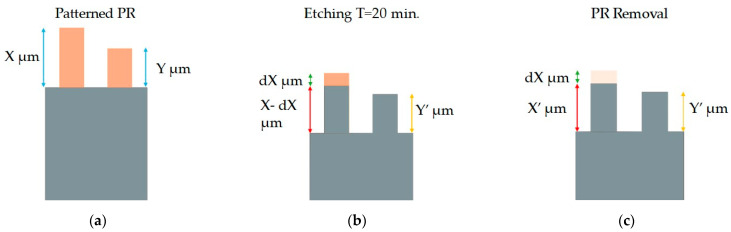
(**a**) The height of the columns is measured. (**b**) Y’ is the etched height of the of column Y. Column X has unetched PR and its total height is measured. (**c**) Removal of PR residues. X’ is measured and dX as a result. Calculated values: selectivity = Y’/Y, Si etch rate = X’/T, PR etch rate = (Χ − dX)/T, dX = X − X’.

To obtain the selectivity dataset, the PR 1225 G was exposed with the mask (test structure B), shown in Figure 2b. Multiple structures were exposed on each wafer and then diced into pieces of 1.5 by 1.5 cm. The samples were developed individually and enough to expose the silicon area between the squares. After development, the height of each square was measured, and then the sample was placed on a Si handling wafer. The sample was held in place by vacuum oil and underwent etching for 20 min. After completion, the squares’ height was measured again. Lastly, since the etching time was not long enough to completely etch away the PR, the remaining PR residue was cleaned with acetone, and the height was measured again. The squares that changed in height were used to calculate the etching rate of PR and Si independently. The procedure is depicted in Figure 3. To calculate the selectivity, all squares were used except the three darkest squares. Since their darkest square PR heights were in the range of 80 to 300 nm, any small deviations in the height after etching, that could be a result of measurement errors or the process itself, would have resulted in vastly altered selectivity compared to the rest of the squares.

For high control in selectivity, and thus, in height scaling, a selectivity chart with minor variation per flow rate was needed. In Set 1, O_2_ flow rate was changed in steps of 1 sccm, from 5 to 15 sccm. In Set 2, SF_6_ was increased in steps of 2 sccm, from 14.5 to 34.5 sccm.

#### 2.3.3. Image Acquisition

We evaluated the performance of the Si Fresnel Lenses using the uncooled LWIR camera STPLUG 612R (Simtrum Pte. Ltd., Singapore) with a detection band of 8–14 µm. Its stock lens was removed, and a custom-designed holder was screwed into the threads of the camera. The holder consists of two parts that screw together. Each of them has a recess (gap) that can fit a rectangular Si piece of up to 2 cm in either direction. The lower part can adjust its distance from the microbolometer, focusing the image on the sensor. Various lenses were tested, ranging in diameter and f#. The images were taken without any bandpass filter, while our lenses were designed without taking into consideration any chromatic aberrations. The formed images were a result of the full LWIR band passing through the lens, thus not displaying the full potential of the lens. Wavelengths outside the operating band of the lens formed erroneous images on the bolometer due to chromatic aberrations, lowering the quality of the final image.

## 3. Results

### 3.1. Dose Linearization of PR and Exposing

**Figure 4:**
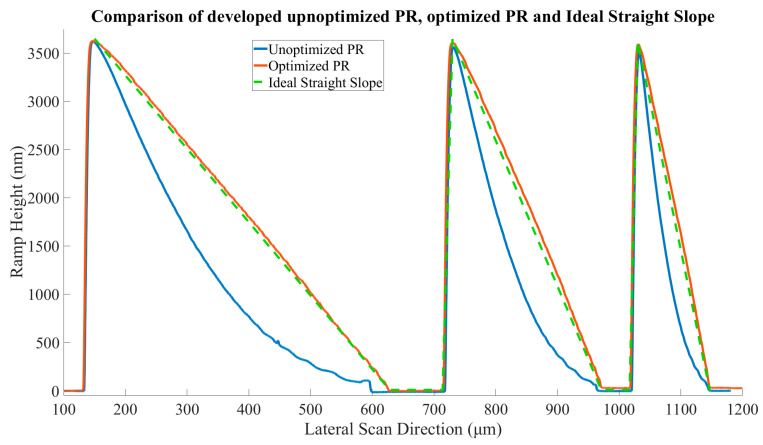
Comparison of PR ramps when their designs are linearized and not. The unoptimized designs show an inward curve in which, in some areas, the difference between desired and actual height can reach values that will severely degrade the image quality. The optimized ramps exhibit excellent linearity.

The evaluation of the dose linearization of PR was performed by comparing the profile of three linear ramps of different widths (500, 250, and 125 µm). The designs were exposed in the same area on the wafer and developed simultaneously. There is a substantial improvement in the behavior of the PR after linearization. Using the profilometer, measurements were conducted and plotted using MATLAB. In Figure 4, the green dotted line represents a theoretical, perfectly linear height profile of PR after exposure and development. 

**Figure 5:**
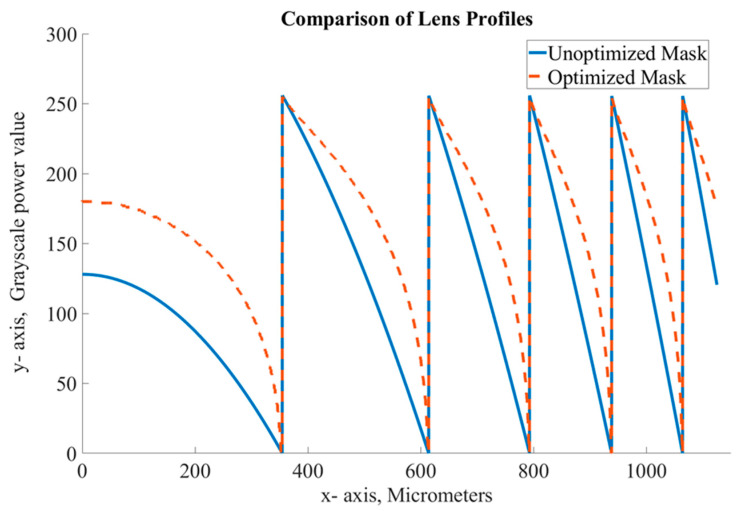
Comparison of the first rings of a Fresnel lens. Each point is adjusted based on the exposure curve resulting on a similar but “scaled” mask.

The calibrated profile of the PR (red line) follows the ideal case very closely. On the other hand, the unoptimized design (blue) lags enough that this mismatch cannot be ignored. The discrepancy between the ideal ramp and the measured uncalibrated ramp can reach in the range of 1 µm for the wider ramp and for the narrowest ramp, it is 1.25 µm, which is a 25% and 31% deviation from the desired profile, respectively. Linearization was also applied to our Fresnel lens case study, altering the GS mask power values accordingly during exposure. A comparison can be seen in Figure 5, where unoptimized and optimized lens designs are compared.

**Figure 6:**
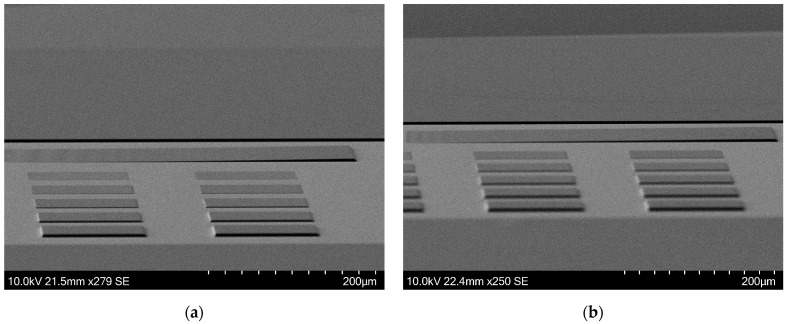
SEM images of the selectivity measuring test structures B in Si. (**a**) On the left, the ramp is unoptimized. From the “shadow” on the sidewalls, it is visible that the ramp lags at gaining height. (**b**) The right optimized ramp starts to gain height earlier than the left one.

Even though the linearization was calculated based on standalone squares of test structure A, it is nicely transferable to structures that are continuous, as evident below. Scanning electron microscope (SEM) images were taken using the Hitachi SU 3500 (Hitachi High-Tech Corporation, Ibaraki, Japan) and shown in Figure 6, which depicts a comparison of the etching test structures. The unoptimized ramp does not gain height according to the mask design and lags compared to the optimized one. The sidewalls of the ramp are visible earlier in the optimized version of the mask, indicating that the ramp begins to gain height early. This behavior is similar to the one observed in Figure 4.

### 3.2. Reactive Ion Etching of Si

#### 3.2.1. Etch Selectivity Control

In these experiments, our objective was to establish an etching process that could provide precise control over selectivity so that the etch height scaling in Si could be as accurate as possible. Both sets of experiments exhibit an almost linear response to the changes in the gas flow under investigation.

**Figure 7:**
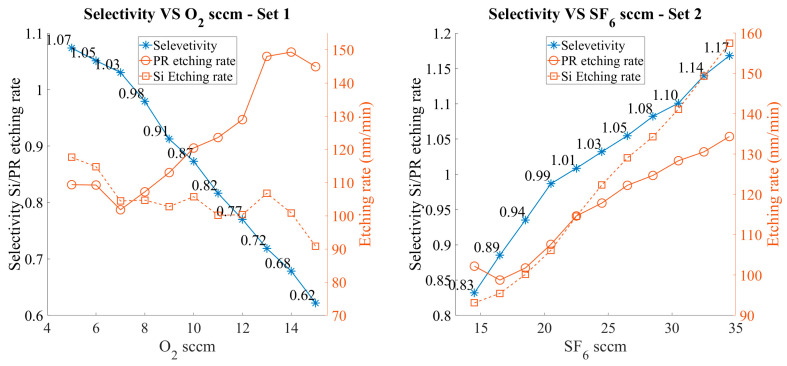
Selectivity and etching rates of Si and PR were examined. The flow rate of SF6 was held constant at 22.5 sccm. As the O_2_ flow rate increased, the etching rate of the RP increased, while the rate of Si decreased slightly. Similarly, with a constant flow rate of O_2_ at 8 sccm and a varying flow rate of SF6, an increase in SF6 flow rate led to an increase in the etching rate of both Si and PR.

In Figure 7, Set 1, the flow rate of O_2_ was varied by 1 sccm. From 5 to 15 sccm, the average selectivity change for each step was 4.5%, with values ranging from 1.07:1 to 0.62:1. Selectivity increments diminished when the O_2_ flow rate was below 8 sccm. The etching rate of PR increased with the increasing flow rate of O_2_, as expected, starting from 109 nm/min to 145 nm/min. The etching rate of Si was slowly reduced, ranging from 117 nm/min to 91 nm/min. The reduction in Si etching rate can be attributed to the congestion of the etchant gases.

In Set 2, the flow rate of SF_6_ was increased in steps of 2 sccm, from 14.5 to 34.5 sccm. Selectivity increments were, on average, 3.4%, ranging from 0.83:1 to 1.17:1. The response to the SF_6_ flow rate was again almost linear. The etching rates of both PR and Si increased in a similar way as selectivity. For PR, the etch rates ranged from 102 nm/min to 134 nm/min, while for Si, they ranged from 93 nm/min to 157 nm/min. The etch rates increase for both materials as SF_6_ etches Si but it physically etches the PR as well.

#### 3.2.2. Accuracy of Pattern Transfer

The evaluation of the quality and accuracy of the Si etching was necessary. Our aim was to evaluate the uniformity of the etching performance across both narrow and wide ramps. To achieve this, a Si wafer was spin-coated with a thick PR 1275 G. Subsequently, the test structure C was exposed, developed, and etched for 1 h. Following these steps, we conducted measurements to determine the heights of the narrowest and widest ramps within the PR layer and the Si substrate. It was observed that the step height of the wider ramps remained consistent, while a marginal decrease of approximately 100 nm in the step height was noted in the narrowest ramp relative to the PR layer. Importantly, this deviation is within an acceptable margin of error.

**Figure 8:**
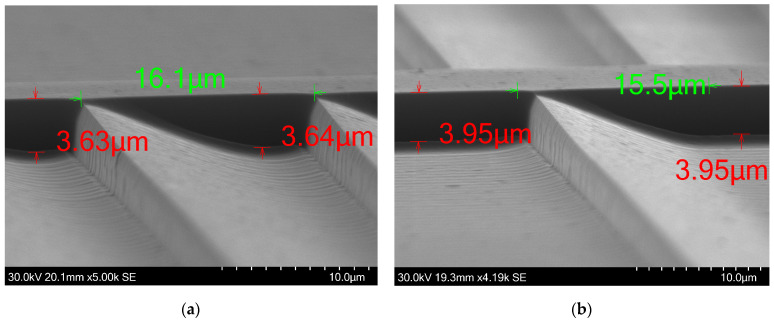
SEM images of part of the test structure C. (**a**) When ramps are placed adjacently, without gaps in between, the slope flattens near the vertical walls. This results in less etched depth. (**b**) When ramps are spaced apart, the etched depth increases by 0.3 μm, reaching closer to the desired etch depth.

**Figure 9:**
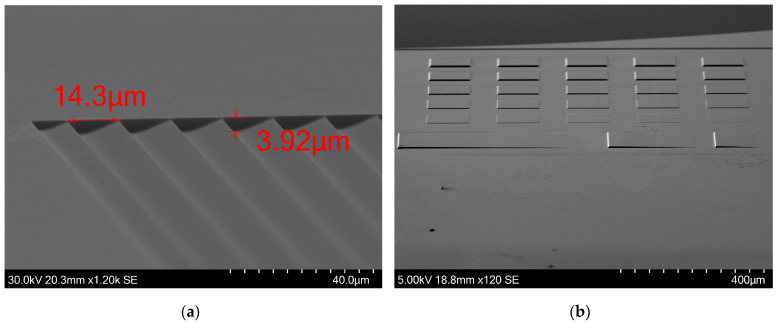
SEM images of the test structures C and B. (**a**) Multiple 14-μm-wide ramps were subjected to etching, resulting in a depth of ~4 μm. (**b**) The etching recipe demonstrated efficacy in etching structures of varying heights. The bottom right square, measuring approximately 80 nm in height, and the top left square, reaching approximately 4 μm in height, were both etched successfully. the 80 nm tall square retained its shape and height despite representing merely 2% of the height of the tallest square.

**Figure 10:**
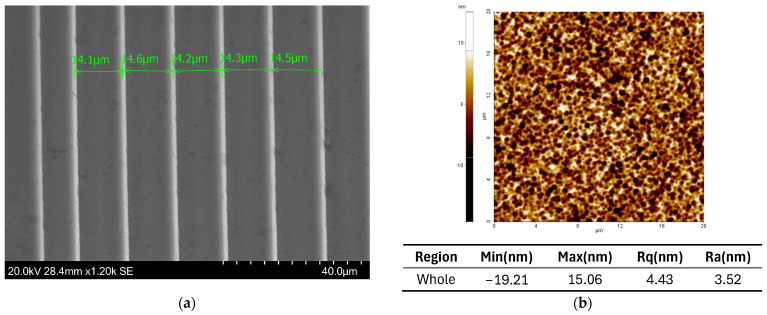
(**a**) SEM images of the last rings of a f#/0.5 Fresnel lens. The widths of the rings are in the range of ~14 μm. (**b**) Using AFM, the surface roughness (Ra) was measured at 3.5 nm. The total area scanned is 400 μm^2^.

We further evaluated the etching performance in areas adjacent to steep height changes. We used the test structure C as it has repetitive height drops with and without gaps between each ramp. The 16-μm-wide ramps were selected due to their dense distribution. It is observed that in areas adjacent to a steep height change, the lower surface flattens, deviating from the intended design profile. When each ramp is separated by some space between nearby ramps, the measured step height averages approximately 3.95 μm. When ramps are not spaced, the maximum measured step height reaches approximately 3.65 μm, as seen in Figure 8. The flattened area experiences a slight increase in height upon reaching the sudden height increase, a feature that is present throughout our fabricated structure. However, the magnitude of this height increase is negligible when the sudden change in etched depth is located within an open area. In Figure 9a, successful fabrication of 14-μm-wide ramps is also demonstrated. Ramps of that width are present in the outer rings of fast Fresel lenses, in the range of f#/0.5, as seen in Figure 10a. The verticality of the steep height changes is good, representing a steep 2π phase jump with a negligible transition zone. In our case study, a Fresnel lens consists of many concentric rings and height jumps occupy a significant area of the lens. The verticality that is shown here is high, avoiding any performance issues from this aspect.

The RIE process is capable of etching both high and short structures successfully. An etched test structure is shown Figure 9b. The shortest square with a height of around 80 nm is present, along with all the taller structures, up to a 4 μm height. With an etching duration of ~45 min, the small square was successfully transferred into the silicon substrate. The pattern was etched into the silicon within a minute, and for the remaining 44 min, it is constantly etched as a Si square, without any PR on top of it. It manages to survive the remainder etch duration, while etching for the rest of the structure is ongoing. Anisotropy was found to be excellent, with the lateral dimensions of the features remaining unchanged.

The average surface roughness was measured utilizing an atomic force microscope (AFM)(XE-200 from Park Systems Co, Suwon, Republic of Korea). The surface roughness value was measured to be 3.5 nm, as depicted in Figure 10b, and is deemed insignificant in terms of performance impairments to the imaging lens, as the operational wavelength is several magnitudes larger than any surface imperfections. Figure 8 also shows the Si surface after etching the 1275 G PR variant. The measurements were obtained by utilizing the built-in software of the SEM. 

### 3.3. Case Study—Fresnel Lens

**Figure 11:**
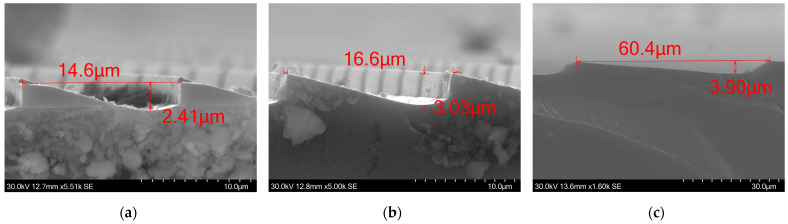
SEM images of rings of different widths, the etched depth varies from (**a**) 2.4 μm, (**b**) 3.0 μm, and (**c**) 3.9 μm. With the increment in the width of the ring, the discrepancy between the resultant and the targeted etch depths diminishes. This phenomenon can be attributed to the non-uniform development of the PR.

We applied the developed GSL process to fabricate Si Fresnel lenses. The results of lenses with and without optimization are presented. They exhibit characteristics mentioned in the previous sections. One of the most important aspects of a Fresnel Lens is to maintain the appropriate 2π step height throughout the surface. In initial fabrication attempts using thin PR, big disparities between experimental results and designs were observed. The step height varied from 2.41 μm for a ramp of width of ~14.6 μm, 3.03 μm for a ramp of width of ~16.6 μm, and 3.9 μm for a ramp of a width of ~60.4 μm, as shown in Figure 11, where the cross-sections of these ramps are depicted. This is a substantial fabrication error that was overcome. This non uniform etching was solved by using the thicker photoresist and developing to a smaller depth of the PR.

**Figure 12:**
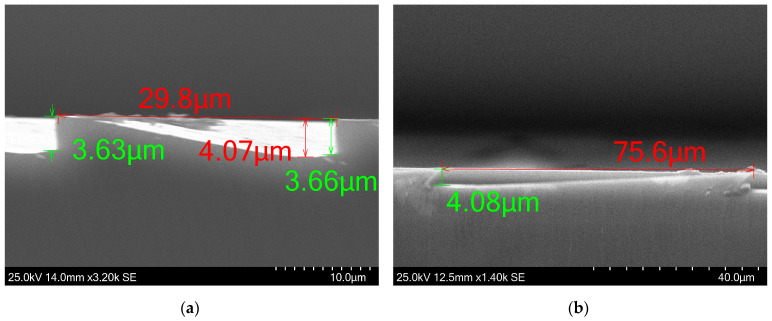
SEM images of rings of a lens with uniform development. (**a**) The cross-section of 29.8-μm-wide ring. The ramp initially flattens and then goes upward, from 4.07 μm to 3.66 μm, identical to the behavior that was observed also in Figure 8. (**b**) The wider rings have achieved the optimal etched depth of 4.08 μm.

**Figure 13:**
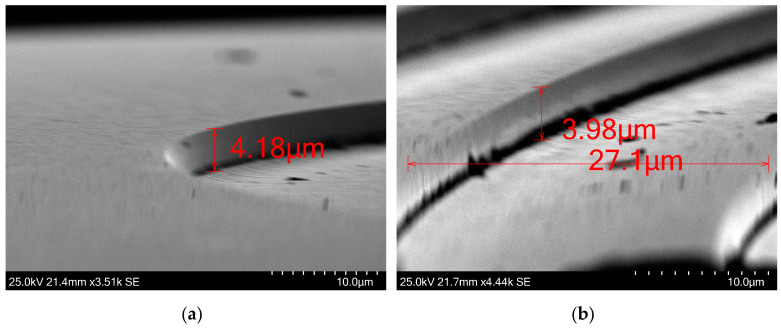
SEM images of inner and outer rings of a lens. (**a**) The central ring achieves a step height of 4.2 μm. The verticality of the step change is excellent. (**b**) For outer rings, the measured step height is 3.9 μm. Both rings are very close to the 4 μm target step height.

Below, in Figure 12, we show that utilizing the test structure C, partially shown in Figure 2 and Figure 8, helped solve this issue and the solution proved to be applicable to the lens structure. The step heights for rings with approximate widths of ~29.8 and ~75.2 μm are found to be identical, at ~4.0 μm. Minor height variations are expected due to the positioning of measuring points. Such small differences shown here are rare instances, and, in most cases, the variance is around 10%, from the central to the outer rings. Figure 13 shows the central ring and an outer ring. The step height change is sharp and narrow, minimizing any potential negative effects it could have on performance.

**Figure 14:**
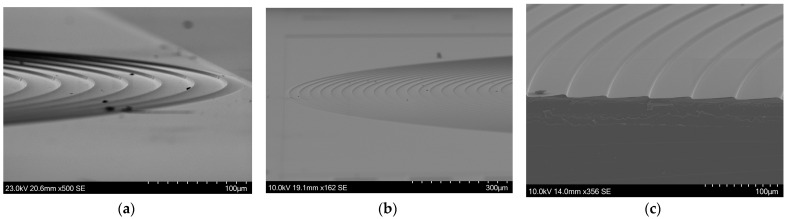
Various SEM images showing Si Fresnel lenses. (**a**) shows the outer rings and (**b**) shows half of a Si Fresnel lens. (**c**) shows the cross-section of a Si lens.

The flattening of the slope that was observed in test structure C was also observed in the cross-sections shown in Figure 11 and less so in Figure 12, as well as the small uphill slope just next to the step height change. This issue remains unresolved. The verticality is similar to what was observed in the test structures. The following SEM images in Figure 14 show larger areas of various fabricated lenses.

**Figure 15:**
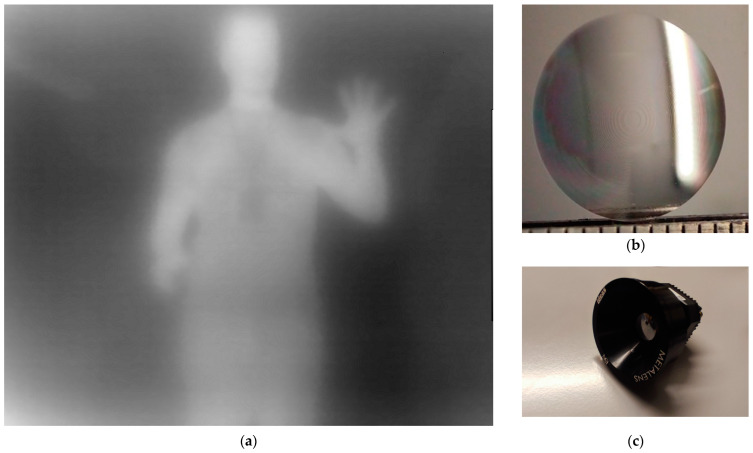
(**a**) An “infrared” image taken (**b**) with the f#/1 and 1.2 cm diameter lens shown on and (**c**) attached to the camera housing using a custom mount as seen in image.

Various fabricated lenses were tested, ranging in diameter, f# and focal length. The widest lens was 2 cm in diameter. Lenses with several combinations of properties were fabricated, with f# as low as f#/0.5 and with various aperture sizes. Figure 15a was taken by a f#/1, 1.2 cm diameter lens (Figure 15b). The lens is held in place using a custom mount and threaded into the LWIR camera (Figure 15c). As the lens phase profile is based on a monochromatic (10 μm) focusing equation, the lack of a bandpass filter hinders the ability to showcase the true performance of the lens. The full LWIR band propagates and forms slightly blurred images at the bolometer surface. The thermal differences between clothing and skin are observable. The lanyard on the chest is distinguishable, as well as the card at the end of it. Facial features are not observable, but the cooler hair is distinguishable.

## 4. Discussion

In this study, results for each step of the fabrication process of GSL are presented. When observing this work from a fabrication point of view, with a non-specific application purpose, it can be seen as a technique to transfer arbitrary 3D patterns into a Si substrate. The non-linear response of the photoresist is the initial issue that one will encounter, which leads to inaccurate PR patterning. Our approach returned results that follow closely the ideal linear response of PR vs. exposure dosage. The uneven development of the Fresnel lens inner and outer rings was resolved by developing the PR pattern to a lesser depth and then scaling to the desired height by choosing a selectivity > 1.0 in the RIE process. Both the central and outer rings now show a variance of around 10% on the rings’ maximum step height, which gives a peak wavefront error of 0.1λ. The RIE process is capable of etching and transferring nano- and micro-sized vertical structures simultaneously into the Si wafer, with a high degree of accuracy when the PR pattern does not have steep changes in height levels. When the pattern is steep, such as in areas near narrow vertical changes in height, the etched Si exhibits a slight flattening of the slope, which needs further investigation as this is a deviation from the desired shape. This could lead to some image degradation and should be resolved in the future to further improve the transferring accuracy of the proposed method. This could be resolved by implementing additional compensations in the mask design and taking into consideration proximity effects that may occur during exposure [30]. For example, a higher exposure dosage on the flat surface can possibly fix this issue. The lateral extent of the steep height changes is quite small, preventing it from affecting the image quality in the case study of the Fresnel lens. Lenses with a 1.2 cm diameter and f#/1 were successfully fabricated, and we displayed images acquired using them. The absence of an achromatic lens solution means that the chromatic aberration present in Fresnel lenses hinders the full potential of the fabricated lens as the full LWIR band (8–12 μm) propagates through the lens and forms a blurred final image on the bolometer sensor.

## 5. Conclusions

The grayscale lithography procedure has been adjusted to meet the needs of the fabrication of high-NA Si Fresnel lenses. Previous published work has tackled some of the issues presented here, but were treated as isolated cases, as mentioned in the introduction. In this work, we treated all issues simultaneously, which was necessary for the successful fabrication of a Fresnel lens. We applied a linearizing calibration to the PR, resulting in very linear ramps. The uneven PR development, which will lead to increased aberrations, was minimized, with a maximum of 10% deviation from the desired height. Ramps with a 14 μm width that are present in f/#0.5 were accurately controlled and fabricated. The etching parameters are capable of providing low increments in selectivity, crucial for scaling the PR height to the correct Si height, while also maintaining very low surface roughness and transfer into Si accurately. Lastly, we applied our approach to fabricate a f/#1 lens with a diameter of 1.2 cm and obtained images with it. The lack of a bandpass filter did not allow for a more accurate representation of the imaging quality of the lens.

An unresolved issue is the flat area that occurs near abrupt changes of PR thickness. This discrepancy is insignificant when they are located in the wider inner ring, as their slope’s angle is very small and the aforementioned area’s height does not change significantly. On the other hand, the angle of the slope of the outer rings is larger and thus the height variation is greater, which results in 10% maximum deviation in height. An improvement is needed to reduce the extent of the flat area in the outermost rings of the Fresnel lens. Excluding this, the final fabricated structures followed the design very closely. The low deviation from the original design is an important step to successfully incorporate the lens with multiple lenses to reduce aberrations.

## Figures and Tables

**Table 1:** The PR is exposed with 160 mJ/cm^2^ and developed for different times. The height difference between rings varies from 0 to 0.2 µm. Further development increased the height difference, making them unsuitable.

Development Duration (s) at Maximum Dose of 160 mJ/cm^2^	14 μm Wide Ramp Height (μm)	140 μm Wide Ramp Height (μm)	Difference (μm)
60	2.6	2.7	0.1
75	3.0	3.0	0
75	2.9	3	0.1
80	3.5	3.7	0.2
95	3.6	3.8	0.2

## Data Availability

Data and images presented in this article are available at https://doi.org/10.23642/usn.26130904.
